# Descriptive study of the consulting and coordination program between Health Center “La Barca” and Jerez Community Mental Health Unit.

**DOI:** 10.1192/j.eurpsy.2023.1897

**Published:** 2023-07-19

**Authors:** A. Torres Laborde, M. Ivaylova Chulkova, A. Bueno Heredia

**Affiliations:** SAS, Cádiz; 2 SAS, Jerez, Spain

## Abstract

**Introduction:**

Patients who receive primary medical care at the Health Center “La Barca” and its corresponding services receive mental health assistance at the Jerez Community Mental Health Unit (CMHU), which belongs to the Clinical Area of Jerez within the structure of the Andalusia Health Service. A psychiatrist from Jerez CMHU is in charge of the consulting and coordination program with Health Center “La Barca”. Any case that may require mental health assistance is brought for discussion at weekly meetings between primary care physicians and the psychiatrist at the health center, with one of the following case resolutions:

- Maintain mental health assistance with primary care physician.
- Refer to the Jerez CMHU for specialized care.
- Single-appointment evaluation and assistance by the psychiatrist within primary care.

**Objectives:**

The aim of this descriptive study was to analyze socio-demographic and clinical characteristics of the population assisted through the consulting and coordination program.

**Methods:**

Socio-demographic and clinical data belonging to the cases brought to the program was collected between 01/06/2018 and 28/02/2020. An *ad-hoc* data collection survey was used for this purpose.

**Results:**

Female/Male 53/23. Mean age: 47.13. Only 20% of the cases discussed were referred for specialized care to Jerez CMHU. 65% of the patients attended the appointments given with the psychiatrist within primary care. The most frequent diagnosis were anxiety disorders, adjustment disorders, and dysthymia.

**Conclusions:**

A significant fraction of the cases discussed at the coordination program are resolved within this framework or through a single appointment with the psychiatrist, implying that the program achieves an important optimization of resources, all the while maintaining high quality healthcare. The data suggests that the consulting and coordination program is an improvement in terms of referral protocols within mental health care. A more detailed study would be necessary to confirm and enhance current data.

**Disclosure of Interest:**

None Declared

